# Cost analysis of in-centre nocturnal compared with conventional hemodialysis

**DOI:** 10.1186/2054-3581-1-14

**Published:** 2014-07-02

**Authors:** Ben Wong, Mark Courtney, Robert P Pauly, Kailash Jindal, Scott Klarenbach

**Affiliations:** Department of Medicine, University of Alberta, Edmonton, Alberta Canada; Institute of Health Economics, Edmonton, Alberta Canada; Alberta Kidney Disease Network, Edmonton, Alberta Canada; Department of Community Health Sciences, University of Calgary, Calgary, Alberta Canada

**Keywords:** Health care costs, Economic analysis, Hemodialysis, In-centre nocturnal hemodialysis

## Abstract

**Background:**

Provision of in-centre nocturnal hemodialysis (ICNHD; 6–8 hours thrice weekly) is associated with health benefits, but the economic implications of providing this treatment are unclear.

**Objective:**

We conducted a health care costing study comparing ICNHD to in-centre thrice-weekly conventional hemodialysis (CvHD).

**Design:**

Micro-costing of both ICNHD and CvHD as practiced at our centre.

**Setting:**

Hemodialysis unit at a tertiary-care hospital in Edmonton.

**Participants:**

An informal survey of 2 other Canadian ICNHD programs was conducted to inform practices that may deviate from ours to guide sensitivity analysis.

**Measurements:**

Resources consumed for each strategy were determined, and the cost of each unit (CAN $2012) was used to calculate incremental costs of ICNHD and CvHD.

**Methods:**

We focused on resources that differ between strategies (staffing, dialysis materials, and utilities). The reference case considered 1:3 staff to patient ratio; alternate scenarios explored nursing pay grade and ratio, full care vs. self-care dialysis (including training costs), and medication costs.

**Results:**

In the reference case, ICNHD was $61 more costly per dialysis treatment compared with CvHD ($9,538 per patient per year). Incremental annual costs for staffing, dialysis materials, and utilities were $8,201, $1,193, and $144, respectively. If ICNHD reduces medication use (anti-hypertensives, bone mineral metabolism medications), the incremental cost of ICNHD decreases to $8,620 per patient per year. In a scenario of self-care ICNHD utilizing a staff-to-patient ratio of 1:10, ICNHD is more costly in year 1 ($15,196), but results in cost savings of $2,625 in subsequent years compared with CvHD.

**Limitations:**

The findings of this cost analysis may not be generalizable to other health care systems, including other parts of Canada.

**Conclusions:**

Compared to CvHD, provision of ICNHD is more expensive, largely driven by increased staffing costs as patients dialyze longer. Alternate staffing models, including self-care ICNHD with minimal staff, may lead to net cost savings. The incremental cost of treatment should be considered in the context of impact on patient health outcomes, staffing model, and pragmatic factors, such as current capacity for daytime CvHD and the capital costs of new dialysis stations.

**Electronic supplementary material:**

The online version of this article (doi:10.1186/2054-3581-1-14) contains supplementary material, which is available to authorized users.

## Abrégé

**Contexte:**

L’hémodialyse nocturne en centre (ICNHD; 6 à 8 heures, trihebdomadaire) est associée à des bienfaits pour la santé, mais nous connaissons mal les répercussions économiques de l’administration de ce traitement.

**Objectifs:**

Nous avons effectué une étude des coûts de revient des soins de santé en comparant l’ICNHD à l’hémodialyse conventionnelle en centre sur une base trihebdomadaire (CvHD).

**Type d’étude:**

Le calcul des coûts individuels de l’ICNHD et du CvHD tel qu’effectué dans notre centre.

**Contexte/Échantillon:**

Le service d’hémodialyse d’un hôpital de soins tertiaires d’Edmonton.

**Participants:**

On a effectué un sondage informel dans le cadre de deux programmes canadiens d’ICNHD afin d’indiquer les pratiques qui pourraient dévier des nôtres, permettant ainsi l’analyse de sensibilité.

**Mesures:**

Les ressources utilisées pour chacune des stratégies étaient déterminées, et le coût de chaque dialyseur (2 012 $CA) a été pris en compte dans le calcul du coût marginal de l’ICNHD et du CvHD.

**Méthodes:**

Nous nous sommes concentrés sur les ressources qui diffèrent selon la stratégie (dotation en personnel, matériel d’hémodialyse et équipements). L’hypothèse supposait un ratio personnel-patients de 1 :3; des scénarios alternatifs examinaient l’échelon de rémunération du personnel infirmier ainsi que les ratios, la prise en charge totale par rapport à l’auto-dialyse (incluant les coûts de formation), et le coût des médicaments.

**Résultats:**

Dans le scénario de référence, l’ICNHD s’est révélé 61 % plus coûteux par traitement de dialyse que le CvHD (9 538 $ par patient par an). Les coûts marginaux de dotation en personnel, du matériel de dialyse et des équipements étaient respectivement de 8 201 $, de 1 193 $ et de 144 $. Si l’ICNHD permet de diminuer l’utilisation de médicaments (antihypertenseurs, médicaments pour le métabolisme minéral osseux), le coût marginal de l’ICNHD diminue à 8 620 $ par patient par an. Dans le cas d’ICNHD en auto-dialyse, qui requiert un ratio personnel-patients de 1 :10, l’ICHND est plus coûteux la première année (15 196 $), mais les économies durant les années subséquentes le rendent comparables au CvHD.

**Limites de l’étude:**

Les conclusions de cette analyse de coûts peuvent se révéler peu valides pour d’autres systèmes de soins de santé, dont ceux d’autres régions du Canada.

**Conclusions:**

Comparativement au CvHD, la fourniture d’ICNHD est plus coûteuse, principalement en raison du personnel supplémentaire requis par des séances de dialyse prolongées. Des modèles de dotation alternatifs, incluant l’ICHND en auto-dialyse qui requiert un minimum de personnel, peut permettre des économies nettes. Le coût marginal du traitement devrait être considéré, en contexte, selon les répercussions possibles sur l’état de santé final des patients, les modèles de dotation, et des facteurs pragmatiques tels que la capacité actuelle du CvHD de jour et les coûts d’investissement de nouvelles stations de dialyse.

## What was known before and what this adds

Provision of in-centre nocturnal hemodialysis (ICNHD; 6–8 hours thrice weekly) is associated with health benefits, but the economic implications of providing this treatment are unclear. Using data from a renal program in Edmonton, Canada, this study examines the incremental costs of ICNHD compared with conventional HD from a health care payer perspective.

## Background

The cost of caring for patients with end-stage renal disease (ESRD) is considerable, largely driven by the provision of chronic dialysis [1]. The majority of ESRD patients are either not suitable for a kidney transplant, or must wait an increasing number of years on dialysis until renal transplantation given the scarcity of available organs. While strategies to increase peritoneal dialysis have been implemented in many jurisdictions, hemodialysis (HD) remains the most common modality, with the majority of those (~80%) receiving conventional thrice-weekly intermittent HD in a satellite or in-centre dialysis unit [2].

There is interest in offering nocturnal HD to patients due to its purported benefits of improved fluid volume and blood pressure control, regression of left ventricular hypertrophy, improved phosphate control, and increased quality of life [3–7]. However, some patients are thought to be unsuitable candidates for a variety of reasons, including inappropriate home environment, lack of social support, inadequate personal resources, or comorbidity precluding self-administration of HD (e.g. severe visual impairment, debilitating arthritis, stroke, etc.). In one centre, ~16% of HD patients were deemed to be appropriate candidates for home nocturnal HD (HNHD) [8]. As such, renal programs are considering in-centre nocturnal HD (ICNHD), typically conducted overnight in an existing in-centre dialysis unit for 6–8 hours thrice weekly. In addition to potential improvements in patient health outcomes and to facilitate patient lifestyle preferences, dialysis providers may also consider ICNHD as a strategy to increase in-centre patient treatment capacity without further expansion of existing HD stations.

Several economic analyses have demonstrated that home HD is either cost neutral or affords cost savings compared to in-centre conventional HD (CvHD), predominantly due to savings associated with nursing care [9–11]; no study has examined ICNHD. To inform rational use of ICNHD, we sought to determine the incremental costs of ICNHD compared with CvHD from a health care payer perspective.

### Study context

#### Program description

The Northern Alberta Renal Program (NARP) oversees and manages renal care in the northern Alberta, Canada with a catchment of approximately 1.7 million. In a pilot program, 12 patients receive ICNHD (6 per HD shift on alternate nights) in an outpatient HD unit at a tertiary-care hospital in Edmonton. This HD unit provides 3 daytime CvHD shifts (18 HD stations per shift, 4 hours per session) with staff-to-patient ratio of 1:3. ICNHD occurs from 2200 to 0600 and the 6 patients are cared for by 2 Registered Nurses (RNs) or 1 RN and 1 Licensed Practical Nurse (LPN). The patients dialyze in beds to facilitate sleep (chairs are removed), and space between stations is increased to allow room for the bed and reduce noise; dividers are also set up to enhance patient privacy. Patients are referred for ICNHD at the discretion of their attending nephrologist, and priority is given to patients with suboptimal ultrafiltration or clearance on CvHD, intra-dialytic hypotension, or lifestyle considerations, including employment or education that conflict with daytime in-centre CvHD; and these patients cannot perform home HD. The duration of ICNHD sessions ranges from 6 to 8 hours, depending on patient preference.

## Methods

Micro-costing methods, comprised of identification, measurement, and valuation of resources, was conducted for ICNHD and CvHD [12]. Identification of relevant health care resources was conducted through iterative discussion with ICNHD nursing staff, NARP financial analyst, clinical engineering, and technical manager overseeing water treatment, focusing on resource use that differs between the two modalities. Identified health care resource categories include staffing, dialysis materials, and utilities (Additional file 1: Table S1). In the reference case, HD machine maintenance, patient-borne costs, and physician billing were equivalent. An informal survey of 2 other Canadian ICNHD programs was conducted to inform practices that may deviate from ours to guide sensitivity analysis.

Staffing costs accounted for salaries and benefits of RNs and LPNs. All other staffing costs, including the salaries of nephrologists, unit clerks, social workers, and other allied health professionals, were assumed equivalent between the two HD modalities. HD materials cost accounted for costs related to dialysis tubing, dialysis needle, dialyzer, dialysate, and bicarbonate solution. To reflect our ICNHD setup, we did not include the cost of setting up and dismantling the HD unit for the nocturnal shift. Depending on the HD unit, additional costs relating to minor renovations may need to be accounted for. Utility costs per treatment factored into costs relating to water and electricity consumption (Additional file 1: Table S1).

Staff (RNs and LPNs) cost per dialysis treatment were calculated on a per hour basis and distributed equally among the patients based on the staff-to-patient ratio for the respective dialysis modality. For materials cost, we tabulated the number of units of the various dialysis supplies consumed per HD session. As accurate water and electricity usage per machine per dialysis treatment for home CvHD and HNHD patients were available, this data was used to inform utility cost. Respective units of measurement for water and electricity consumption per HD session were captured in liters and kilowatt-hours.

The constituent costs were summed to arrive at total cost per HD session for both ICNHD and CvHD, and subsequently, calculated on a per patient-year basis. All costs were expressed in 2012 Canadian dollars.

Scenario analyses were also conducted to reflect other practices in Canada. A scenario of self-care ICNHD (with both a 1:6 and a 1:10 staff-to-patient ratio) was assessed, where patients receive training during the initiation of ICNHD and then perform machine setup and takedown, as well as access cannulation, with either no or reduced nursing assistance. Training costs (inflated to CAN $2012), arising from 3.65 weeks of training, were obtained from a micro-costing study of patients enrolled a randomized-controlled trial (RCT) that compared HNHD and CvHD [11]. The additional cost of patient training, $17,821 per patient, which accounts for nursing time, capital costs, and equipment per patient, is added to the cost of providing ICNHD in the first year. A scenario exploring the effect of nursing grade mix (50% RNs vs. 100% RN) on incremental costs was also explored.

Difference in medication costs for ICNHD patients versus CvHD patients was examined. We used results from the Alberta RCT [11] (which used units of medications and formulary prices inflated to CAN $2012) to inform medication costs of anti-hypertensive agents, bone mineral metabolism medications, and erythropoietin-stimulating agents (ESAs) for those on CvHD. Medication costs for ICNHD patients were informed by the Alberta RCT (which showed cost reduction in anti-hypertensive agents and bone mineral metabolism medications only) and other observational studies reporting on medication use in ICNHD vs. CvHD, which showed cost reduction in all 3 medication classes [13–18].

ICNHD may be considered as an alternate strategy to creating new dialysis units and attendant capital costs when existing patients cannot be accommodated. A scenario analysis was performed to explore possible incremental costs. We assumed a scenario in which there is a surplus of HD patients in the context of an existing HD unit functioning at full capacity. All patients would then either be accommodated by ICNHD, or be treated with CvHD in a newly built in-centre dialysis unit. We explored the effects of size (4, 6, 9 stations) and capacity (25%, 50%, 75%, 100%) of the new HD unit. Capital costs of establishing new HD units were based on estimates from NARP.

## Results

In the reference case (1:3 staff-to-patient ratio with nursing grade of 50% RN), ICNHD was $61 more costly per dialysis treatment compared with CvHD (Table 1, Additional file 2: Table S2). Difference in staffing cost was responsible for greater than 85% of the incremental cost; materials and utility during ICNHD accounted for 13% and 2% respectively. Incremental annual costs per dialysis patient for staffing, dialysis materials, and utilities were $8,201, $1,193, and $144, respectively for ICNHD vs. CvHD. Overall, ICNHD was $9,538 more per patient-year (Table 1).

**Table 1:** **Absolute and incremental costs of ICNHD and CvHD**

Cost categories	Absolute ICNHD cost ($)	Absolute CvHD cost ($)	Incremental cost ($, ICNHD - CvHD)
**1. Materials per treatment**	43.49	35.84	+ 7.65
**2. Staff per treatment**	111.38	58.81	+ 52.57
**3. Utilities per treatment**	2.73	1.81	+ 0.92
**Total cost per treatment per patient**	157.60	96.46	+ 61.14
**Total annual cost per patient**	24,585.86	15,048.28	+ 9,537.58

ICNHD: in-centre nocturnal hemodialysis; CvHD: conventional hemodialysis.

Accounting for plausible reduction in anti-hypertensive agents and bone mineral metabolism medications for ICNHD patients compared to CvHD patients, the cost differential between the two HD modalities is reduced from $9,538 to $8,620 per annum (Table 2). If we also account for reduction in ESA requirement (suggested in observational studies, but not shown in RCTs), the cost differential further reduces to $6,119 per annum.

**Figure 1:**
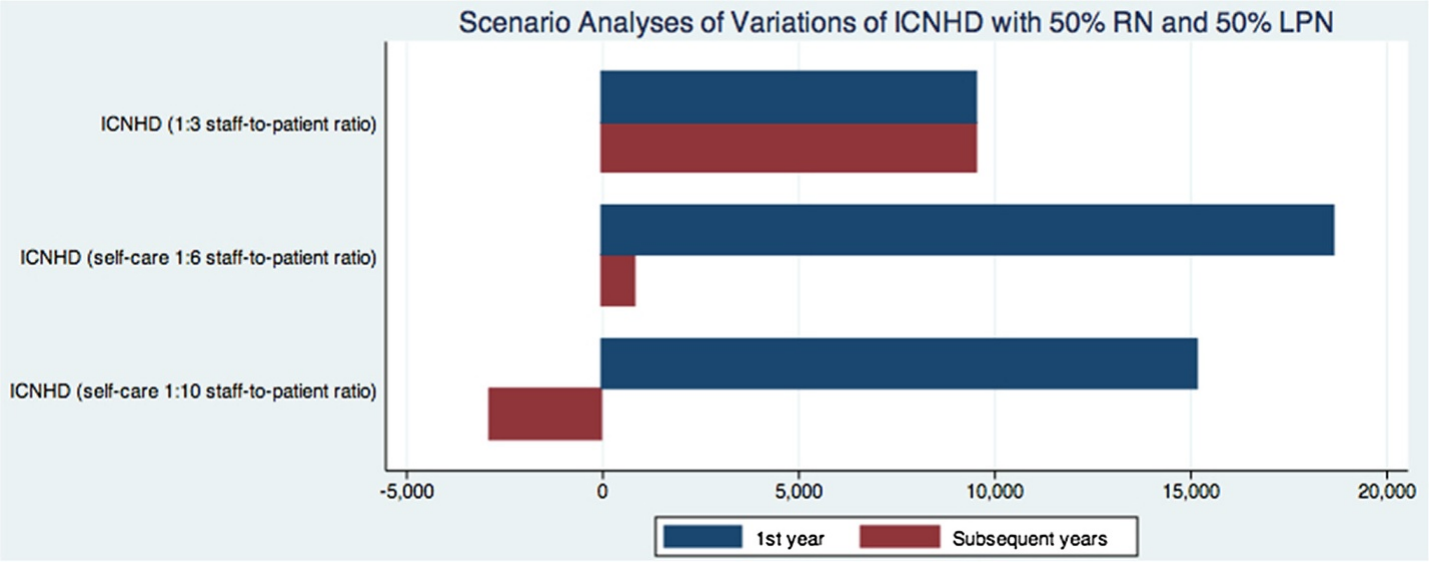
**Scenario analyses of alternate staff-to-patient ratio with 50% RN and 50% LPN.** Costs are incremental compared with CvHD. ICNHD, in-centre nocturnal hemodialysis; CvHD, conventional hemodialysis; RN, registered nurse; LPN, licensed practical nurse.

Scenario analyses of alternate staff-to-patient ratio and nursing grade of 50% RN are presented in Figure 1. Incremental costs are similar with 100% RN (figure not shown). A self-care ICNHD program with a 1:10 staff-to-patient ratio and a nursing grade of 50% RN resulted in an annual incremental cost of $15,196 (vs. $9,538 in the reference case) in the patient’s first year of dialysis and annual saving of $2,625 in subsequent years (Figure 1).

**Table 2:** **Impact of difference in medication requirement on relative costs between ICNHD and CvHD**

Resource category	CvHD ^1^ ($)	ICNHD ($)	Δ Cost ($, ICNHD – CvHD)
**Medication**			
Anti-hypertensives	756.00	378.00	(−378.00)
ESAs	6,252.00	3751.20	(−2,500.80)
Bone mineral metabolism medications	1,080.00	540.00	(−540.00)
**Reference – no difference in medication use (annual cost/patient)**	**15,048.28**	**24,585.86**	**+ 9,537.58**
**Reduced anti-HTN and bone mineral metabolism medications only (annual cost/patient)**	**16,884.28**	**25,503.86**	**+ 8,619.58**
**Reduced in all medication categories (annual cost/patient)**	**23,136.28**	**29,255.06**	**+6,118.78**

^1^annual cost of CvHD was informed from the Alberta randomized-controlled trial [11].

Assumptions:

- 50% reduction in number of anti-hypertensives required based on observational studies [13–17].

- 40% reduction in required ESA dose based on observational studies [16–18].

-50% reduction in amount of bone mineral metabolism medications required based on observational studies [13, 15, 16, 18].

ICNHD: in-centre nocturnal hemodialysis (7 hour sessions, 3x /week, 1:3 staff-to patient ratio); CvHD conventional hemodialysis (4 hour sessions, 3x /week, 1:3 staff-to-patient ratio); ESAs: erythropoiesis-stimulating agents.

Incremental costs of constructing a new HD unit vs. providing ICNHD is shown in Table 3. A new HD unit would be required to operate for close to 10 years at near-full capacity before the initial capital costs equilibrate with the increased operating costs of ICNHD (relative to CvHD). This was consistent across various sizes and capacities of the new HD unit.

**Table 3:** **Estimated incremental costs comparing construction of new dialysis unit vs. ICNHD (ICNHD – CvHD)**

Size of new HD unit added	Capacity of added HD unit			
	25%	50%	75%	100%
**4 stations (capital cost ~ $2,000,000)**				
1 year	$1,942,775	$1,885,549	$1,828,324	$1,771,098
5 years	$1,713,873	$1,427,745	$1,141,618	$855,490
10 years	$1,427,745	$855,490	$283,236	(−$289,019)
**6 stations (capital cost ~ $2,700,000)**				
1 year	$2,614,162	$2,528,324	$2,442,485	$2,356,647
5 years	$2,270,809	$1,841,618	$1,412,427	$983,236
10 years	$1,841,618	$983,236	$124,853	(−$733,529)
**9 stations (capital cost ~ $4,000,000)**				
1 year	$3,866,474	$3,742,485	$3,608,959	$3,484,971
5 years	$3,332,369	$2,712,427	$2,044,796	$1,424,853
10 years	$2,664,739	$1,424,853	$89,592	(−$1,150,293)

Assumptions:

- all patients can be accommodated by ICNHD or would be treated with CvHD in a newly built in-centre dialysis unit of varying station capacity.

- annual operating cost per ICNHD patient $24586; annual operating cost per CvHD patient $15048 (incremental cost $ x annually).

- capital costs of establishing new HD units based on estimates from the Northern Alberta Renal Program.

- capacity refers to the use of stations within the unit, assuming 3 shifts per day, 6 days per week.

ICNHD: in-centre nocturnal hemodialysis; CvHD: conventional hemodialysis; HD: hemodialysis.

## Discussion

In this cost analysis of ICNHD, we have demonstrated that provision of ICNHD cost approximately $9500 more per annum per patient relative to CvHD. This is primarily attributed to increased staffing costs of RNs and LPNs from both longer duration of dialysis and increased wage rates for overnight shifts. An alternate scenario of self-care ICNHD that utilized a higher nursing staff-to-patient ratio substantially reduce the incremental cost, with some scenarios demonstrating cost savings after the first year with ICNHD.

Self-care ICNHD program with a higher nursing staff-to-patient ratio (1:6 and 1:10) offers an attractive alternative from a health payer perspective. This variation of ICNHD is already operational in other centres (Michael Copland, personal communication, March 28, 2013). Patients are trained the same way as for HNHD patients; they are expected to be competent with all aspects of HD care, except for machine maintenance. While ICNHD, compared to CvHD, can potentially lead to net cost savings, a concern exists in that it may detract from patients choosing HNHD, for which operating costs are likely lower and there is stronger evidence of clinical benefits [4, 5]. Accordingly, providers should be encouraged to first consider HNHD and offer patients the opportunity to transfer to HNHD after they have completed their training for self-care ICNHD.

A pragmatic rationale for ICNHD is patient numbers exceeding existing in-centre HD unit capacity. While increasing the number of patients on home dialysis therapies may offset this to some degree, the majority of dialysis patients are likely to require in-centre HD. If the capacity of existing in-centre HD units were exceeded, either expanding an existing HD facility or constructing a new facility would be required. A previous study showed that the cost of renovating existing space for a six-station satellite unit is approximately $250,000 to $300,000 Canadian [19], while the cost of constructing a new HD facility consisting of 6 to 9 stations is estimated to be $2.5-3.5 million Canadian. Our scenario analysis shows that implementing ICNHD to maximize use of existing infrastructure may be advantages given the very large capital costs of creating a new dialysis unit, despite the greater incremental treatment costs of ICNHD relative to CvHD. If the incremental treatment costs can be further reduced, as by employing a self-care ICNHD program, ICNHD becomes even more attractive. These estimates should be approached with caution, as the true capital cost of a new HD unit are not known with certainty, may vary by setting and jurisdiction, and may be modified if the unit is de novo or is utilizing currently unused space. Further, the decision to create new units is also influenced by other factors, including geographical considerations and anticipated future growth of the HD population,

ICNHD may be more attractive if other benefits are realized. Although the clinical benefits of ICNHD have not been rigorously evaluated, published literature suggests clinical improvements are achieved. ICNHD improves blood pressure and phosphate control, with concomitant reductions in requirements of anti-hypertensives and phosphate binders compared with CvHD [6, 13–17, 20–26]. Reduced need for ESAs to achieve similar levels of hemoglobin has also been found [13, 15–17, 21], although not in RCTs; we have accounted for savings from reduced ESA requirements for interest only as current evidence does not support a reduction in use. Some studies have also suggested reduced mortality and hospitalization rates for ICNHD patients compared to CvHD patients [14, 17, 18]. However, the clinical benefits associated with ICNHD do not necessarily imply causation. To-date, there has been no RCTs evaluating the benefits of ICNHD relative to other HD modalities; most studies are either cohort studies or before-after studies that are subject to the limitations with respect to internal validity. As such, we have used potential cost savings in scenario analyses, and have not conducted a comprehensive economic evaluation that considers health consequences, including cardiovascular events, and survival.

There have been few studies evaluating the quality of life of ICNHD patients [13, 22, 25, 27]. This precludes the feasibility of conducting cost utility studies in this area at present. However, there are potential productivity gains afforded by ICNHD. ICNHD allows patients to maintain full-time employment that would otherwise not be possible with CvHD; it may also allow patients to receive training for independent home HD in conjunction with their HD treatments overnight, while maintaining employment in the day. Future studies in ICNHD should include quality of life measures and assessment of the effect on patients’ employment.

Our study has important strengths. Many economic evaluations have previously been performed on various forms of intensive HD therapy, but to our knowledge, never with respect to ICNHD. Using a micro-costing technique, we have formally identified the incremental costs of ICNHD compared with CvHD. However, we also acknowledge several limitations with this study. First, cost comparisons between ICNHD and CvHD were assumed to be equivalent with regards to HD machine maintenance, patient-borne costs, overhead, and physician billing. Potential cost differential in these areas may be missed, but may not be important given that differences in staffing costs between the two HD modalities contributed to a large proportion of the overall cost differential. Second, in our scenario analysis, the cost of patient training for self-care ICNHD may be underestimated. The relatively short training period was informed by the Alberta RCT, where a large proportion of patients who were established on HNHD enrolled in this trial [4]. The average duration of training may be higher in reality; in NARP, new home HD patients typically require 6 weeks of training. However, the training cost accounted for a dedicated RN performing patient training/teaching (in addition to another nursing staff caring for the patient on dialysis), thus potentially overestimating the staffing component of training costs; our estimate of overall training costs may be a reasonable approximation. Third, our estimates of the relative reduction in medication requirements for ICNHD patients were based on findings from a few cohort and quasi-experimental studies [13–18]. The precise effect would require more rigorous investigations, but we used conservative estimates in our analysis. Finally, the findings of this cost analysis may not be generalizable to other health care systems, including other parts of Canada. Differences in physician billings, salary structures of RNs and LPNs, and construction costs of new HD units may result in findings different than those identified here.

## Conclusions

In conclusion, we found that, compared to CvHD, provision of ICNHD is more costly, largely driven by increased staffing costs related to longer patient dialysis time. Alternate staffing models, including self care ICNHD, results in net cost savings. The cost of treatment should be considered in the context of impact on patient outcomes, staffing model, and other factors, such as current available capacity for in-centre HD.

## Electronic supplementary material

Additional file 1: Table S1: Components of the Differential Cost Between ICNHD and CvHD, Unit of Measurement, Valuation Method, and Valuation Sources. (DOCX 66 KB)

Additional file 2: Table S2: Constituent Differential Costs of ICNHD and CHD. (DOCX 64 KB)
