# Supplementary material for: Cost analysis of in-centre nocturnal compared with conventional hemodialysis
Source: Can J Kidney Health Dis. 2014 Jul 2;1:14. doi: 10.1186/2054-3581-1-14 (PMC4349597; doi:10.1186/2054-3581-1-14)
Supplement: Supplementary file 1 — Additional file 1: Table S1: Components of the Differential Cost Between ICNHD and CvHD, Unit of Measurement, Valuation Method, and Valuation Sources. (DOCX 66 KB) [file 40697_2014_14_MOESM1_ESM.docx]

| **Cost Categories** | **Definition** | **Unit of Measurement** | **Valuation Sources** | **Metric** |
| --- | --- | --- | --- | --- |
| Nursing | Salary and benefits for RNs and LPNs | Number of work hours relative to staff-to-patient ratio | Salaries of RNs and LPNs were collected from respective collective agreements between the RN and LPN unions, and the Province of Alberta;  mid-scale base salaries for the respective profession were used; hourly rates include base salary, base benefits, shift differential, and charge nurse differential | Costs per HD session per patient |
| Materials | Dialysis tubing, dialysis needle, dialyzer, dialysate, and bicarbonate solution | Number of dialysis tubing, dialysis needle, dialyzer, jugs of dialysate, and jugs of bicarbonate solution consumed | Materials costs were obtained from an electronic hospital-purchasing catalog, with prices determined by the vendor | Costs per HD session |
| Utility Use | Costs relating to water and electricity consumption | Number of liters of water consumed, number of KwH of electricity consumed | Estimates were made using the consumption required by the HD and portable reverse osmosis machines used by patients, factoring into the time required for machine priming, takedown, and disinfection | Costs per HD session |
