# Supplementary material for: Cost analysis of in-centre nocturnal compared with conventional hemodialysis
Source: Can J Kidney Health Dis. 2014 Jul 2;1:14. doi: 10.1186/2054-3581-1-14 (PMC4349597; doi:10.1186/2054-3581-1-14)
Supplement: Supplementary file 2 — Additional file 2: Table S2: Constituent Differential Costs of ICNHD and CHD. (DOCX 64 KB) [file 40697_2014_14_MOESM2_ESM.docx]

| Dialysis Modality | Resource | Unit of Measurement | Cost per Unit |
| --- | --- | --- | --- |
| ICNHD | Staffing  RN  Base pay  Shift differential (2300 to 0700)  Charge pay  Benefits  LPN  Base pay  Shift differential (2300 to 0700)  Benefits | 7 hours  7 hours  7 hours  7 hours  7 hours  7 hours  7 hours | $40.39 per hour  $5 per hour  $2 per hour  $8.08 per hour  $29.17 per hour  $5 per hour  $5.83 per hour |
|  | Materials  Bellco tubing  Needle  Rexeed 15L dialyzer  Dialysate  1250g bottle of bicarbonate solution | 1 arterial line  1 venous line  2 – 15G backeye needles  1 dialyzer  2 jugs  1 bottle | $5.5 per line  $5 per line  $0.69 per needle  $15.5 per dialyzer  $4.5 per jug  $7.11 per bottle |
|  | Utility  Water  Electricity | Reverse osmosis reject @ 1.2 L/minute, 100 L of water for setup, 100 L of water for takedown  1 hour setup time, 1 hour takedown time | 1.42 per m^3^  5.94 kWh for first hour + 1.98 kWh for each additional hour |
| CvHD | Staffing  RN  Base pay  Shift differential (1500-2300) – assuming the patient dialyzes from 1300 to 1700  Charge pay  Benefits  LPN  Base pay  Shift differential (1500-2300) – assuming the patient dialyzes from 1300 to 1700  Benefits | 4 hours  2 hours  4 hours  4 hours  4 hours  2 hours  4 hours | $40.39 per hour  $2.75 per hour  $2 per hour  $8.08 per hour  $29.17 per hour  $2.75 per hour  $5.83 per hour |
|  | Materials  Bellco Tubing  Needle  Rexeed 15L dialyzer  Dialysate  720g bottle of bicarbonate solution | 1 arterial line  1 venous line  2 – 15G backeye needles  1 dialyzer  1 jug  1 bottle | $5.5 per line  $5 per line  $0.69 per needle  $15.5 per dialyzer  $4.5 per jug  $3.96 per bottle |
|  | Utility  Water  Electricity | Reverse osmosis reject @ 1.2 L/minute, 100 L of water for setup, 100 L of water for takedown  1 hour setup time, 1 hour takedown time | 1.42 per m^3^  5.94 kWh for first hour + 1.98 kWh for each additional hour |
